# Association between risk of oral precancer and genetic variations in microRNA and related processing genes

**DOI:** 10.1186/1423-0127-21-48

**Published:** 2014-05-17

**Authors:** Roshni Roy, Navonil De Sarkar, Sandip Ghose, Ranjan R Paul, Anindita Ray, Indranil Mukhopadhyay, Bidyut Roy

**Affiliations:** 1 Human Genetics Unit, Indian Statistical Institute, 203, B.T. Road, Kolkata 700108, India; 2 Oral Pathology Department, Guru Nanak Institute of Dental Science and Research, 157/F Nilganj Road, Kolkata 700114, India

**Keywords:** Oral leukoplakia, miRNA, DNA sequence variation, MDR, Gene expression

## Abstract

**Background:**

MicroRNAs have been implicated in cancer but studies on their role in precancer, such as leukoplakia, are limited. Sequence variations at eight miRNA and four miRNA processing genes were studied in 452 healthy controls and 299 leukoplakia patients to estimate risk of disease.

**Results:**

Genotyping by TaqMan assay followed by statistical analyses showed that variant genotypes at *Gemin3* and *mir-34b* reduced risk of disease [OR = 0.5(0.3–0.9) and OR = 0.7(0.5–0.9) respectively] in overall patients as well as in smokers [OR = 0.58(0.3–1) and OR = 0.68(0.5–0.9) respectively]. Among chewers, only *mir29a* significantly increased risk of disease [OR = 1.8(1–3)]. Gene-environment interactions using MDR-pt program revealed that *mir29a*, *mir34b, mir423* and *Xpo5* modulated risk of disease (p < 0.002) which may be related to change in expression of these genes as observed by Real-Time PCR assays. But association between polymorphisms and gene expressions was not found in our sample set as well as in larger datasets from open access platforms like Genevar and 1000 Genome database.

**Conclusion:**

Variations in microRNAs and their processing genes modulated risk of precancer but further in-depth study is needed to understand mechanism of disease process.

## Background

Leukoplakia is one of the major forms of oral precancerous lesions that can be clinically characterized primarily as a non-scratchable white lesion of the oral mucosa [1]. Incidence of this lesion varies from 1.3 to 2.1 per 1000 individuals in different parts of India depending on the types of tobacco habits but prevalence of this lesion was found to be about 17 per 1000 tobacco users [2]. Several factors like tobacco carcinogen, alcohol, HPV infection and genetic predisposition are suspected causative agents for leukoplakia [3]. A survey on Indian populations found that ~80% of oral cancers were preceded by pre-cancerous lesions and oral leukoplakia being most common among them [4].

MicroRNAs (miRNAs) are highly conserved small regulatory RNAs that play fundamental biological roles in all known plant and animal species [5]. They are transcribed mainly by *RNA Pol II* and go through series of processing before they become functionally active. Initial large primary transcripts or “pri-miRNAs” are sequentially cleaved in the nucleus by microprocessor complex which contains *DROSHA* ribonuclease and *DGCR8*, to form premature precursors or pre-miRNA. These 70–80 nt long precursors are exported to cytoplasm by *RAN-GTPase* and *Exportin 5* (*XPO5*), after which they are further cleaved to produce ‘mature’ (~22 nucleotides in length) single-stranded miRNAs that are bound to the RISC complex comprising *DICER, GEMIN3, AGO1* etc. MiRNAs are reported to function by binding ‘target’ mRNAs through partial or complete base-pair complementarity at 3′UTR and this leads to either a decreased rate of polypeptide synthesis or complete degradation of mRNA templates, respectively [6,7]. To date, more than 2500 miRNA molecules have been identified in the human genome and they play important roles in a broad range of physiological and pathological processes [8-10]. Genetic variation at miRNA and their processing genes have potential to affect regulation of multiple cellular pathways instrumental in cancer development and susceptibility [11]. In order to investigate their potential role in altering risk of oral leukoplakia, single nucleotide polymorphisms (SNPs) in four miRNA processing and pri-miR and pre-miR regions of eight miRNA genes were studied in leukoplakia patients. Then, gene-environment interaction was studied using Multi Dimensionality Reduction (MDR) approach to understand risk of leukoplakia. We also examined whether expression of these genes are modulated by SNPs not only in our samples but also in publicly available databases, such as Genevar and 1000 Genome project.

## Methods

### Patients and controls

This study was approved by “Review committee for protection of research risk to humans, Indian Statistical Institute”. Unrelated controls (n = 452) and oral leukoplakia patients (n = 299) were recruited from two tertiary referral hospitals, namely R. Ahmed Dental College and Hospital [12] and Gurunanak Institute of Dental Science and Research, Kolkata, India [13]. All patients and controls had at least one kind of tobacco habit. Patients were referred to these hospitals for biopsy and diagnosis by primary health centers and were not under any prior medication for leukoplakia. Unrelated controls visited these hospitals for treatment of dental ailments, not related to any kind of precancer and cancer, and had no family history of cancer. Demographic details and tobacco habits were noted after written consent.

### Sample collection and processing

Venous blood (~3 ml) was collected from all patients and controls and stored at −20 ºC until DNA isolation. Genomic DNA was isolated by salt extraction method [14] and QIAamp DNA extraction kit (QIAGEN, Valencia, CA). Biopsy tissues collected from patients were used for histopathology and expression study. Normal tissues were also collected from few individuals who were recruited as controls. To study RNA expression, part of the biopsy tissues were collected in RNA Later and stored at −20°C until use. Total RNA was extracted using *All Prep DNA/RNA Mini Kit* (QIAGEN, Valencia, CA) following protocol provided with the kit.

### SNP selection

All 12 SNPs selected in this study have been implicated in various tobacco related squamous cell carcinomas in previous reports [15-18]. Eight of them: rs11614913(*C/T*) at *miR-196a2*, rs2910164 (*G/C*) at *miR-146a*, rs7372209 (*C/T*) at *miR-26a-1*, rs6505162 (*A/C*) at *miR-423*, rs213210 (*A/G*) at *miR-219-1*, rs2660304 (*G/T*) at *miR-137*, rs2187473 (*T/C*) at *miR-34b* and rs24168 (*A/G*) at *miR-29a* are located at miRNAs. Remaining four SNPs are located in miRNA processing genes: rs197412(*C/T*) at *GEMIN3*, rs3742330 (*A/G*) at *DICER1,* rs11077 (*A/C*) at *XPO5* and rs14035(*C/T*) at *RAN*[15,16].

### Genotyping

Genotyping at all SNPs was performed by Taqman method in 7900HT FAST Real-Time PCR system (Applied Biosystems, USA). To reconfirm genotypes determined by Taqman method, ~15% of the samples were randomly selected and further genotyped by a different person (who had no prior information of the genotypes) either by re-sequencing (ABI 3100 Genetic Analyzer, Applied Biosystem, USA) or PCR-RFLP. Except for *mir146a* and *mir196a-2*[19], primers for sequencing and RFLP studies were designed by us.

### Gene expression

Expressions of miR processing genes *(Gemin3 and Xpo5)* and miRNAs *(mir29a, mir34b* and *mir423)* in 19 leukoplakia and 19 control tissues were studied by TaqMan method (7900HT Fast Real Time PCR System, Applied Biosystem, USA). Normalized expression of a gene was calculated as ΔCt with respect to endogenous control genes, *RNase P* or *RNU44* in case of miR processing or miRNA genes respectively*.*

### Statistical analysis

Each SNP data from controls was checked for Hardy-Weinberg equilibrium (HWE). Male/female distribution and number of smoker/chewers were different in control and leukoplakia groups. So, age-, sex- and tobacco dose-adjusted risk of leukoplakia was calculated as odds ratios (ORs) with 95% confidence intervals (CIs) for all genotypes by binary logistic regression analysis using PLINK software [20]. Risk of leukoplakia was also calculated in patients stratifying them on basis of their tobacco habits. Chi-square test with Yates’ correction, when necessary, was used for comparison of genotype proportions. Three different genetic models for disease, such as, dominant model (comparing homozygous major genotype with variant allele-carrying genotypes), recessive model (comparing major allele-carrying genotypes with homozygous variant genotype) and additive model (i.e. trend test) were considered in analysis. Since there were multiple SNPs involved in the study, multiple testing corrections were done using Benjamini-Hochberg method [21]. Spearman’s correlation test was performed to evaluate difference between gene expressions depending upon genotypes at SNPs.

### Multi dimensionality reduction (MDR) analysis

To analyze possible interaction among SNPs and other covariates, non-parametric MDR approach was used [22,23]. All 12 SNPs and covariates (Pack Year and Chewing Year) were considered to construct interaction models in patients comparing with those of control individuals. For every SNP, three genotypes were coded as 0, 1 and 2; no-smoking/chewing dose was coded as 0, low smoking/chewing dose (<mean PY or CY in controls) coded as 1 and high smoking/chewing dose (>mean PY or CY in controls) was coded as 2. Statistical significance was determined using permutation testing in MDRpt (version 1.0_beta_2). Ten fold cross-validation and 1000 fold permutation testing were used and interaction models with p-value <0.05 were considered as significant. Cross-validation consistency (CVC) score is a measure of degree of consistency with which identified interaction is most evident among all possibilities [24]. Among significant models, cross validation consistency (CVC) ≥ 9 was considered as important, as the data was cross validated 10 times by MDR. Best model was then defined with largest testing balance accuracy (TBA) among important models. Testing Balanced Accuracy (TBA) is a measure of degree to which interaction accurately predicts case–control status, taking into account the ratio of cases to controls, with scores between 0.50 (indicating that model predicts no better than chance) and 1.00 (indicating perfect prediction). Scores of at least 0.55 are considered ‘interesting’ [24].

### Analysis of 1000 Genome database

Small RNA expression data and whole genome sequencing data on 485 individuals of 1000 Genome project has been recently released and is openly accessible [25]. MiRNA RNA-Seq and genotype data, obtained from lymphoblastoid cell line, of 87 Caucasian was extracted for our analysis. MiRNA RNA-Seq data was analyzed using miRExpress software [26] which converts available FASTQ data into read counts. Read counts were normalised using two methods- i) RPM or Reads per million [27] and ii) DEseq tool from Bioconductor software [28]. Genotype data were obtained from 1000 Genome web browser.

### Analysis of data from Genevar platform

Genevar (GENe Expression Variation) platform has gene variation and gene expression data from HapMap3 population (http://www.sanger.ac.uk/resources/software/genevar/) [29]. After launching the application in a Java enabled computer, gene name and *rs* ID is entered in the “eQTL-SNP-Gene” option. Genevar provides spearman’s correlation coefficient and p value for analysis between gene expression and genotypes for 8 different populations.

## Results

Controls as well as patients were recruited from same dental hospitals and were ethnically similar. Although both smokers and chewers are equally affected by leukoplakia, but abundance of smokers (85%, including exclusive smokers and mixed habitués) in the leukoplakia group is corroborated by the high number of males (88%) in leukoplakia patients (Table 1). Analysis of genotypes in control samples showed that population was in HWE (p =0.08 to 1.0 for all SNPs). There was 100% concordance between genotypes determined by different methods. Histopathologically, 73% of leukoplakia tissues had mild dysplasia while remaining 27% had moderate dysplasia.

**Table 1:** Demography and tobacco habits of leukoplakia patients and controls

**Subjects and tobacco habits**	**Control [N = 452] (%)**	**Leukoplakia [N = 299] (%)**	**p-value**
Male	367(81)	263(88)	**0.01**
Female	85(19)	36(12)	
Age (Mean ± SD)	48.5 ± 11	47.4 ± 10.4	0.18
Exclusive smokers^a^	175(39)	170(57)	**<0.001**
Exclusive chewers^b^	185(42)	44(15)	**<0.001**
Mixed habitués^c^	92(19)	85(28)	**0.01**

^a^Exclusive smokers have tobacco smoking habit only.

^b^Exclusive chewers have tobacco chewing/dipping habit only.

^c^Mixed habitués have both tobacco smoking and chewing habits.

Minor allele homozygote at *Gemin3* reduced risk of leukoplakia at both allelic and genotypic level (p = 0.04 and 0.03 respectively) (Table 2). Genotypes containing minor allele at *mir-34b* also reduced risk of leukoplakia (p = 0.05). When stratified on basis of tobacco habits, *Gemin3* and *mir-34b* reduced risk of leukoplakia among smokers too [p = 0.05 and 0.04 respectively] while minor allele homozygous genotype at *mir-29a* increased risk of leukoplakia significantly [p = 0.05] among chewers. But none of the associations remained significant after multiple testing corrections. Combined effect of risk genotypes at *Gemin3* and *mir34b,* was also estimated on the basis of their weighted O.R. [13]. It was found that combination of *TT* at *Gemin3* and *CT* or *TT* at *mir34b* together reduced risk of leukoplakia [OR = 0.7 (0.5–0.9), p = 0.02].

**Table 2:** Polymorphisms at miRNA and miRNA processing genes and risk of leukoplakia (Only SNPs with significant p-values are shown)

**Gene (SNP)**^**c**^	**Genotype and Alleles**	**Control (%)/leukoplakia (%)**	**O.R (95% C.I)**^**a**^	**p- value**
*Gemin3(rs197412)*	*TT + CT*	389(87)**/**273(92)	Ref	**0.03**
	*CC*	58(13)**/**25(8)	0.5(0.3–0.9)	
	*T*	580(62)**/**417(70)	Ref	**0.04**
	*C*	314(38)**/**179(30)	0.7(0.6–0.9)	
*mir34b(rs2187473)*	*CC*	153(35)**/**116(41)	Ref	**0.05**
	*CT + TT*	286(65)**/**166(59)	0.7(0.5–0.9)	
	*C*	527(60)**/**357(63)	Ref	0.29
	*T*	351(40)**/**207(37)	0.8 (0.7–1.1)	
**Within Tobacco Stratified Individuals**				
(^b^Tobacco Smokers : Controls- 267/Leukoplakia-255)				
*Gemin3(rs197412)*	*TT + CT*	222(86)/230(92)	Ref	**0.05**
	*CC*	35(14)/21(8)	0.58(0.3–1)	
*mir34b(rs2187473)*	*CC*	80(32)/98(41)	Ref	**0.04**
	*CT + TT*	169(68)/140(59)	0.68(0.5–0.9)	
(^b^Tobacco Chewers : Controls-277/Leukoplakia-129)				
*mir29a(rs24168)*	*GG + AG*	228(84)/78(74)	Ref	**0.05**
	*AA*	44(16)/27(26)	1.8(1–3)	

^a^adjusted for age, sex and tobacco habits.

^b^Smokers/chewers include exclusive smokers/chewers along with mixed habitués.

^c^*Gemin3*- Dominant model; *mir34b* and *mir29a*-Recessive model (with respect to risk allele).

Few individuals could not be genotyped at certain loci.

Fourteen factors [12 SNPs along with tobacco smoking (PY) and/or chewing dose (CY)] were considered in MDR analysis but maximally six factors were taken for interaction. Three-, four-, five- and six-factors gene-environment interaction models were identified and model/s with an average cross-validation consistency (CVC) of 9 out of 10 and p value of <0.05 in MDR permutation testing program (MDR-pt) were considered to be significant (Table 3). Three significant models were observed: one model comprised PY, CY and *mir196a2* with Testing Balance Accuracy or case–control status prediction accuracy of 67% (p < 0.001), second model consisted of PY, CY, *mir34b* and *mir29a* with prediction accuracy of 67% (p < 0.001) and lastly PY, CY, *mir29a, mir34b, mir423* and *Xpo5* with prediction accuracy of 62% (p < 0.002). So, gene-environment interaction seems to modulate risk of leukoplakia.

**Table 3:** MDR interaction analysis between loci and tobacco habits in “leukoplakia vs control” group

**Best predictive interactive model**^**a**^	**TBA**^**b**^	**CVC**^**c**^	**1000 permutation p value**^**d**^
PY CY *mir196a2*	0.67	9	**<0.001**
PY CY *mir34b mir29a*	0.67	10	**<0.001**
PY CY *mir29a mir34b mir423 Xpo5*	0.62	9	**<0.002**

^a^Best model was selected as one with minimum prediction error, maximum CVC and significant p-value after 1000 permutation.

^b^TBA corresponds to testing balanced accuracy, the accuracy with which the model can predict disease status.

^c^Cross Validation Consistency: calculated out of 10.

^d^p value calculated after running 1000 permutations in MDR-pt program.

**Figure 1:**
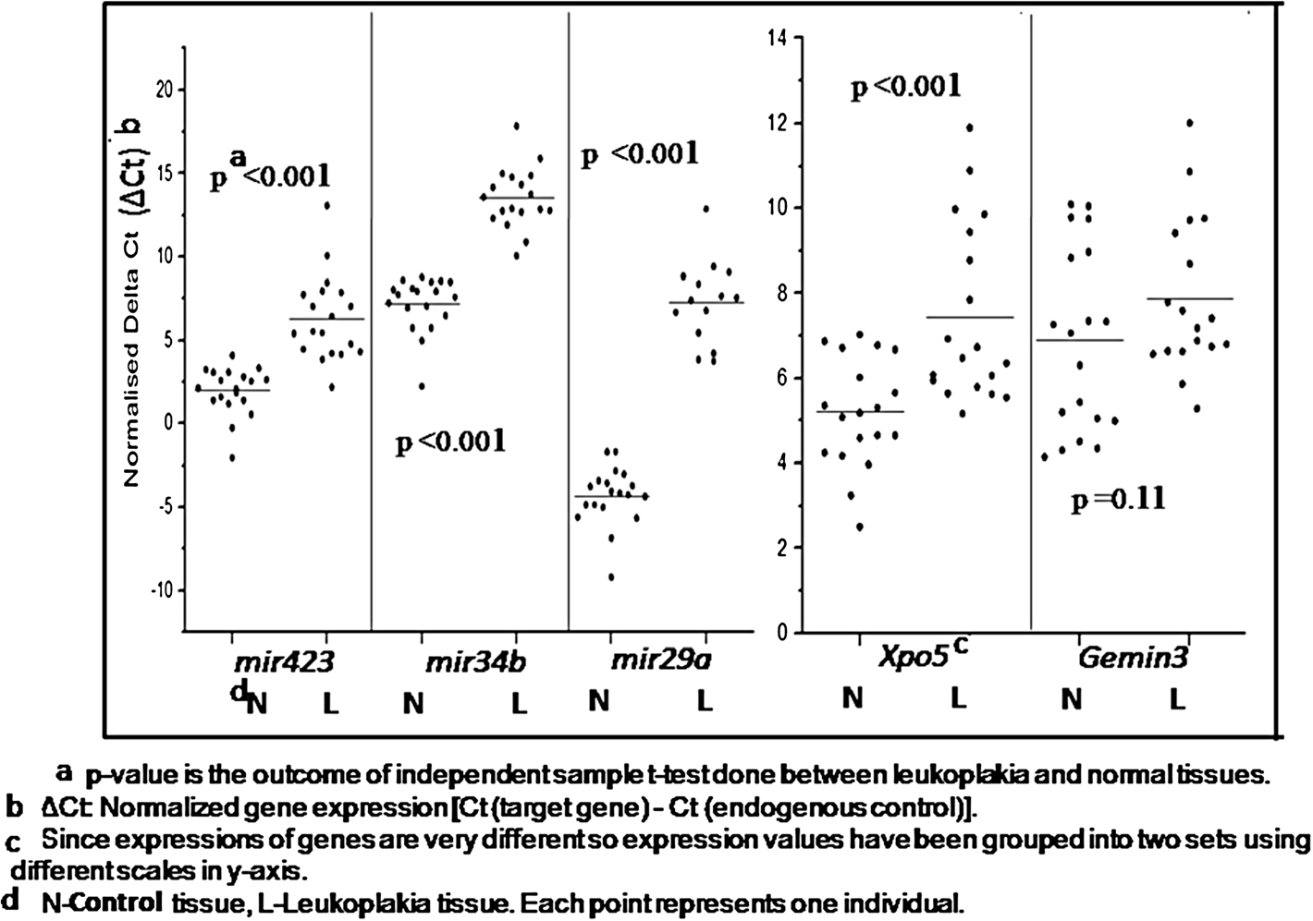
Comparison of gene expression of three microRNAs and two microRNA processing genes in leukoplakia tissues with respect to control tissues from different individuals.

In order to compare expression of *Gemin3, Xpo5, mir34b, mir29a, mir423* on the basis of genotypes, we calculated ΔCt value in leukoplakia tissues with respect to endogenous gene (Ct_gene of interest_ – Ct _*RNASEP* or *RNU44*_). On performing Spearman’s correlation test between ΔCt values and genotypes of a gene, no significant association was observed (p value = 0.28–0.81) (data not shown). Again, after analyzing freely available miR-RNASeq data generated by 1000 Genome, no significant association was observed between expression and genotypes at SNPs of significant miRNAs by performing correlation test (p = 0.2–0.9 in DEseq normalized data, p = 0.3–0.8 in RPM normalized data). For *Gemin3* and *Xpo5,* we also checked available eQTL-SNP-Gene analysis between genotypes at SNP and gene expression for CEU population in Genevar platform which contains genotype and expression data of HapMap 3 population. But, we did not get any significant difference in expression across genotypes (p value = 0.11 and 0.16 respectively). But interestingly, when we compared expression of these miRNA and processing genes between leukoplakia and “control” tissues, we found significant difference in expression (Figure 1).

## Discussion and conclusion

Variant homozygote at *Gemin3* reduced risk of leukoplakia significantly (OR = 0.5, 95% CI = 0.3–0.9). Few other studies had also reported association between oral premalignant lesion and SNP at *Gemin3*[15,30,31]. This polymorphism did not seem to alter *Gemin3* expression in our small dataset of 19 leukoplakia tissues or larger Hapmap3 data set. This SNP lies in the exonic region and, thus, may alter gene activity or could be in the vicinity of other causal SNPs. Study by Clague et al. [15] has shown that SNPs *rs197412* at *Gemin3, rs7372209* at *mir26a-1* and *rs3742330* at *Dicer1* altered risk of oral pre-malignant lesion but except *Gemin3*, our study could not find association between polymorphisms at *mir26a-1* and *Dicer1* and risk of leukoplakia (data not shown). It is worth mentioning that sample size was smaller (136 patients and 136 controls) in the report by Clague et al. [15]. Variant allele containing genotypes at *mir34b* were found to reduce risk of leukoplakia in overall sample population (p = 0.05) as well as in smokers (p = 0.04) (Table 2). Again, we did not find any difference in expression of *mir34b* across its three genotypes at *rs2187473*. But this SNP lies 1 kb downstream of *rs4938723* that has been shown to lie in the promoter region of *mir34b* and influence risk of various types of cancer [32-34]. So, it needs to be investigated whether these two SNPs are in LD. In our recent study on cancer patients, we got similar results for *Gemin3* and *mir-34b*[13]. It is interesting to study how these two polymorphisms alter risk of both oral precancer and cancer. It was found that risk of leukoplakia was reduced significantly (p =0.02) when patients harbored protective variant genotypes at both loci (*CC* at *Gemin3* and *CT/TT* at *mir34b*).

In order to analyze implication of polymorphism on gene expression, we not only studied our sample set (N = 19) but also explored publicly available databases like 1000 Genome and Genevar platform. We assumed that if a SNP alters gene expression, it would do so in all cells including lymphoblastoid cell line. Although, the sample size was in the range of 87–100 in databases, we still did not find any significant variation in expression across genotypes at a SNP, similar to results in our sample set (data not shown).

Alongside we used MDR to predict statistical epistasis which describes an effect exceeding the combined effects of each genetic factor [35]. It has been observed that there might be non-linear interaction between tobacco habits and *mir29a, mir34b, mir423* and *Xpo5* and, as a result, risk of leukoplakia may be modulated (Table 3). So, we compared expression of these genes in leukoplakia with respect to control tissues to understand whether there is any deregulation in expression independent of polymorphism. Although expression of *mir29a, mir34b, mir423* and *Xpo5* was not modulated by genotypes at SNPs but expression of these genes was significantly different in leukoplakia with respect to control tissues. This finding further emphasizes importance of these miRNAs in leukoplakia development. In a recent report, *miR-29a* was found to be down regulated in serum of patients with high risk oral lesions (HRL), showing high specificity in identifying HRLs [36]. They also observed that *miR-423* was differentially expressed across oral squamous cell carcinoma, carcinoma in situ, and controls. *MiR-423* was found to significantly promote cell growth and cell cycle progression at the G1/S transition in HCC cells [37]. Functional studies have reported both up- and down- regulation of *miR-34b* in tongue and oral squamous cell carcinoma, respectively [17,38]. *MiR-34b* is also involved in a number of tumorigenesis-related molecular mechanisms, including epithelial-mesenchymal-transition (EMT) [39]. So, it suggests that our observation related to *mir29a, mir34b, mir423* and *Xpo5* in leukoplakia might have important implications in transformation of normal epithelium to leukoplakia.

There are several reports on association of microRNA SNPs with a wide repertoire of disease [11,40] but most of them did not show how these variations actually alter miRNA expression or activity. Here, it is shown that SNPs at miRNA and processing genes are associated with risk of leukoplakia statistically but most of the associations are lost after correction for multiple testing. First limitation of the study is small number of candidate SNPs so it is important to re-examine association study with more number of SNPs since it is known that a large number of miRNAs are associated with risk of different precancer and cancer. Second limitation is sample size of leukoplakia patients. Partially, this might be due to the fact that many precancer patients prefer to avoid hospital since precancer is not life-threatening. But it is always worthwhile to study larger number of patients. Importance of this study lies in the observation that expression of genes, which interact with environmental factors to modulate risk of leukoplakia, was significantly altered in leukoplakia compared to control tissues (Figure 1). Since miRNAs target genes involved in cell proliferation, apoptosis etc., it is essential not only to study expression of more miRNAs involved in these pathways but also explore significance of more miRNA-associated polymorphisms on large sample sets to validate their significance in leukoplakia.

## Competing interests

The authors declare that they have no competing interest.

## Authors’ contributions

RR and BR concepted the study, designed experiment and wrote manuscript. SG and RRP helped in sample collection and histopathology reports. RR, NDS and AR involved in laboratory experiments. NDS and IM helped in statistical analysis of data. All authors have read and approved the final manuscript.
